# Evaluating smartphone-based 3D imaging techniques for clinical application in oral and maxillofacial surgery: A comparative study with the vectra M5

**DOI:** 10.1007/s10006-024-01322-2

**Published:** 2025-01-10

**Authors:** Robin Hartmann, Maximilian Weiherer, Felix Nieberle, Christoph Palm, Vanessa Brébant, Lukas Prantl, Philipp Lamby, Torsten E. Reichert, Jürgen Taxis, Tobias Ettl

**Affiliations:** 1 Present Address: Department of Oral and Maxillofacial Surgery, University Hospital Regensburg, Franz-Josef-Strauß-Allee 11, 93053 Regensburg, Germany; 2 Department of Computer Science, Chair of Visual Computing, Friedrich-Alexander-Universität Erlangen-Nürnberg, Cauerstraße 11, 91058 Erlangen, Germany; 3 Regensburg Medical Image Computing (ReMIC), Ostbayerische Technische Hochschule Regensburg (OTH Regensburg), Galgenbergstr. 32, 93053 Regensburg, Germany; 4 Regensburg Center of Biomedical Engineering (RCBE), OTH Regensburg and Regensburg University, Galgenbergstr. 32, 93053 Regensburg, Germany; 5 University Center of Plastic, Aesthetic, Hand and Reconstructive Surgery, University Hospital Regensburg, Franz-Josef-Strauß-Allee 11, 93053 Regensburg, Germany; 6 Department of Plastic, Aesthetic, Hand and Reconstructive Surgery, Hospital Passau, Teaching Hospital of the University of Regensburg, Innstraße 76, 94032 Passau, Germany

**Keywords:** Three-dimensional Surface Imaging, Smartphone-based Surface Imaging, TrueDepth, Stereophotogrammetry, Oral and Maxillofacial Surgery

## Abstract

**Purpose:**

This study aimed to clarify the applicability of smartphone-based three-dimensional (3D) surface imaging for clinical use in oral and maxillofacial surgery, comparing two smartphone-based approaches to the gold standard.

**Methods:**

Facial surface models (SMs) were generated for 30 volunteers (15 men, 15 women) using the Vectra M5 (*Canfield Scientific, USA*), the TrueDepth camera of the iPhone 14 Pro (*Apple Inc., USA*), and the iPhone 14 Pro with photogrammetry. Smartphone-based SMs were superimposed onto Vectra-based SMs. Linear measurements and volumetric evaluations were performed to evaluate surface-to-surface deviation. To assess inter-observer reliability, all measurements were performed independently by a second observer. Statistical analyses included Bland–Altman analyses, the Wilcoxon signed-rank test for paired samples, and Intraclass correlation coefficients.

**Results:**

Photogrammetry-based SMs exhibited an overall landmark-to-landmark deviation of M = 0.8 mm (SD =  ± 0.58 mm, n = 450), while TrueDepth-based SMs displayed a deviation of M = 1.1 mm (SD =  ± 0.72 mm, n = 450). The mean volumetric difference for photogrammetry-based SMs was M = 1.8 cc (SD =  ± 2.12 cc, n = 90), and M = 3.1 cc (SD =  ± 2.64 cc, n = 90) for TrueDepth-based SMs. When comparing the two approaches, most landmark-to-landmark measurements demonstrated 95% Bland–Altman limits of agreement (LoA) of ≤ 2 mm. Volumetric measurements revealed LoA > 2 cc. Photogrammetry-based measurements demonstrated higher inter-observer reliability for overall landmark-to-landmark deviation.

**Conclusion:**

Both approaches for smartphone-based 3D surface imaging exhibit potential in capturing the face. Photogrammetry-based SMs demonstrated superior alignment and volumetric accuracy with Vectra-based SMs than TrueDepth-based SMs.

## Introduction

Three-dimensional (3D) surface imaging is widely employed in oral and maxillofacial surgery (OMFS), in which precise assessments of anatomically complex structures and subtle volumetric changes are critical [1–3]. The technology is utilized in numerous clinical contexts, improving patient care and communication between patients and clinicians in pre- and postoperative settings [4–11]. Therefore, 3D surface imaging has become a leading technology, gradually replacing conventional photography in surgical planning and outcome evaluations [12].

Recently, smartphone-based approaches for 3D surface imaging have been introduced [13–21]. Despite these technological advancements, smartphone-based 3D surface imaging is still not sufficiently integrated into standard procedures in OMFS.

Few studies have evaluated the capability of smartphones to capture anatomically complex facial regions. Interestingly, some studies highlight the potential of smartphone-based methodologies for capturing facial features with cost-effectiveness and portability, while other studies report limited clinical applicability. D’Ettorre et al. evaluated facial surface models (SMs) of 40 individuals, utilizing three different systems: the 3dMDtrio stereophotogrammetry system (*3dMD Inc., USA*), the iPhone XS with the TrueDepth-based Bellus3D Face application (*Bellus3D Inc., USA*), and the iPhone XS with the application Capture (*Standard Cyborg Inc., USA*). The research documented the duration of image acquisition and processing, and also gauged the surface-to-surface deviation and distance between 18 landmarks on the reference images from 3dMD and those obtained with Bellus3D or Capture [13]. The authors concluded that the use of smartphone applications in conjunction with the TrueDepth camera demonstrates promising results. According to the authors, the primary benefits lie in cost-effectiveness and portability. Andrews et al. compared SMs of the face captured with the 3dMDface system and the iPhone 11 Pro TrueDepth camera combined with the Bellus3D Face application. They found that 97% of landmarks were within 2 mm of error compared to the reference data. The authors reported an overall root mean square (RMS) difference between the iPhone 11 Pro and 3dMD system of 0.86 mm ± 0.31 mm. High intra-observer and inter-observer reliabilities were reported [16]. Seifert et al. performed a study involving 15 patients to compare the accuracy of three 3D facial scanning applications for the iPhone 14Pro (EM3D *(Brawny Lads SoftwareUSA)*, Polycam *(Polycam Inc., USA),* and ScandyPro *(Scandy LLC., USA)*) with a stationary photogrammetry system (3dMD). They found that the smartphone applications demonstrated mean surface deviations of 1.46 mm for EM3D, 1.66 mm for Polycam, and 1.61 mm for ScandyPro. A mean landmark-to-landmark deviation of 1.27 mm for Polycam, 1.26 mm for ScandyPro, and 1.45 mm for EM3D was observed. The authors concluded that smartphone-based systems offer a cost-effective and portable alternative to stationary systems, particularly in resource-limited settings [20]. Nightingale et al. compared facial SMs of 20 participants acquired using the Apple iPhone 8S (*Apple Inc., USA*) in conjunction with the Camera + 2 iOS application (*tap tap tap LLC., USA*) and the Artec Spider* (Artec Group, Luxembourg)* structured light scanner. They found an accuracy of 1.3 mm ± 0.3 mm between Artec-based and smartphone-based SMs. They concluded that smartphone-based photogrammetry is a reliable, low-cost alternative for clinical 3D facial imaging [21]. In contrast, Thurzo et al. observed differences between SMs exceeding 3 mm when comparing SMs generated by the TrueDepth camera utilizing the Bellus3D Dental Pro application and SMs generated by cone beam computed tomography [17]. They concluded that smartphone-based 3D surface imaging has limited clinical relevance. Nevertheless, they recommended that employing smartphone-based 3D surface imaging for facial assessments, especially under circumstances where precision below 3 mm is not imperative, could still yield value.

While the studies present a thorough approach, it is crucial to note that due to technological advancement, the applicability of smartphone-based 3D surface imaging may have improved. Additionally, detailed volumetric assessments of the face were performed by few prior investigations.

To address this obstacle, this study was aimed at clarifying the applicability of smartphone-based 3D surface imaging for clinical use in OMFS by comparing two smartphone-based approaches with the established gold standard, the Vectra M5 system (*Canfield Scientific, USA*). SMs generated by the two approaches were subsequently compared based on their alignment with the Vectra M5 system employing landmark-to-landmark distance analyses and volumetric assessments. This comparative analysis aims to provide insights into the potential clinical use of smartphone-based 3D surface imaging and contributes to a broader understanding of its accuracy in comparison to established technologies in the field of OMFS.

## Material and methods

### Study protocol

This prospective monocentric study was conducted at the Department of Oral and Maxillofacial Surgery, University Hospital Regensburg, Germany, following approval from the local ethics committee (23–3400-101). The investigation involved 30 healthy adult students enrolled at the University of Regensburg, excluding individuals with recent craniofacial surgery, maxillofacial trauma, or significant skeletal deformities.

### Participant preparation

Consistent with prior studies on 3D surface imaging, participants were positioned in a standardized posture under controlled lighting conditions. After receiving an explanation of the procedure, they were seated on a stool and instructed to maintain a neutral facial expression while keeping their heads in a natural position. Participants were also directed to wear a hairband and to remove any makeup. Following the protocol by Othman et al., 15 specific landmarks were identified on each participant’s face using a white eyeliner [22].

**Fig. 1:**
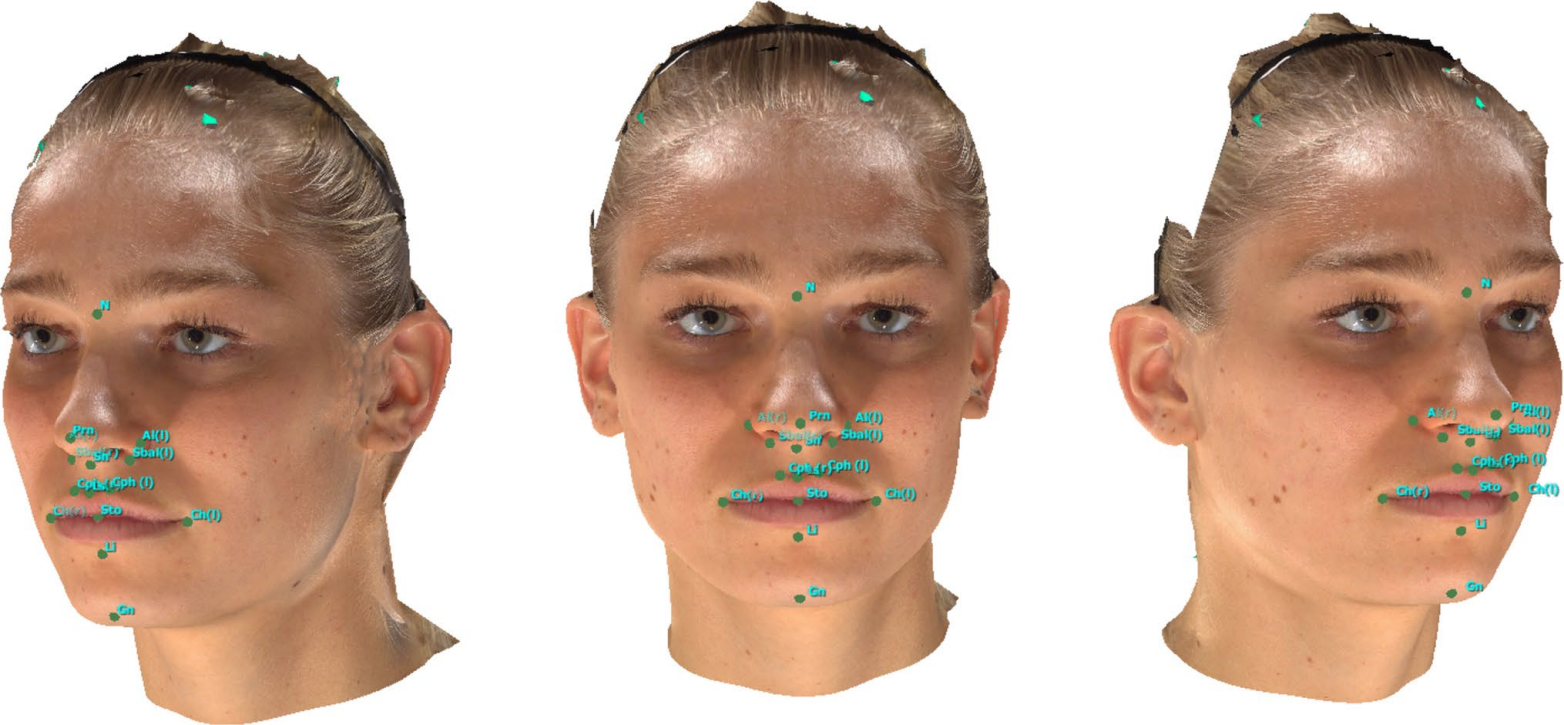
Landmarks: Appearance of a 20-year-old female participant; the iPhone 14 Pro using the “3D-Scanner App” V2.1.2 was utilized to create the SM; (1) soft tissue nasion (N), (2) pronasale (PRN), (3) subnasale (SN), (4) labrale superius (LS), (5) stomion (STO), (6) labrale inferius (LI), (7) soft tissue gnathion (GN), (8) alare (AL) (L), (9) alare (AL) (R), (10) subalare (SBAL) (L), (11) subalare (SBAL) (R), (12) christa philtri (CPH) (L), (13) christa philtri (CPH) (R), (14) cheilion (CH) (L), (15) cheilion (CH) (R)

Figure 1 provides an overview of all landmarks.

### 3D data acquisition

The study design included obtaining 3D SMs of each participant’s face using three different methods: stereophotogrammetry with the Vectra M5, the smartphone application “3D-Scanner App” V2.1.2 (*Laan Consulting Corp., USA*) utilizing the TrueDepth camera of the iPhone 14 Pro (*Apple Inc., USA*), and the light detection and ranging (LiDAR) camera of an iPhone 14 Pro in conjunction with photogrammetry. The smartphone application was selected, based on its capability to offer both a “TrueDepth-Mode” and a “Photo-Mode”.

While the TrueDepth camera employs vertical-cavity surface-emitting laser (VCSEL) technology to directly generate a metric point cloud, which is later used for SM generation, in the photogrammetry setup, the iPhone’s LiDAR sensor is utilized to ensure a metrical 3D reconstruction. LiDAR employs time-of-flight measurements to ascertain the distance (i.e., depth) between an object and the sensor [23].

The stereophotogrammetry-based Vectra M5, renowned for its high accuracy and widely employed in 3D facial imaging, was utilized as a reference in the study [22, 24–26]. Organizing multiple photographs into stereo pairs and integrating their overlapping regions to create a 3D SM, stereophotogrammetry is considered the gold standard for 3D surface imaging [19, 27]. The Vectra M5 uses the Vectra Analysis Module (VAM) for SM analysis [28].

**Fig. 2:**
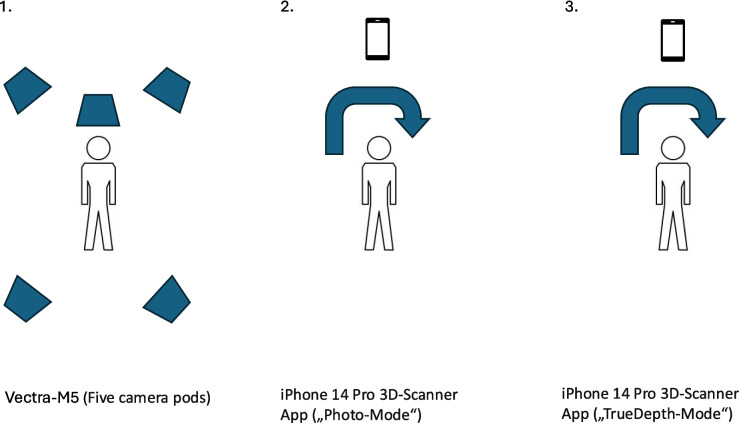
*Comparative visualization of 3D imaging techniques:* SMs generated using three methods: (1) Vectra M5, featuring five camera pods mounted on a rigid frame for stereophotogrammetry, (2) iPhone 14 Pro “3D Scanner App” V2.1.2 in “Photo-Mode”, which required capturing images in a circular motion, and (3) iPhone 14 Pro “3D Scanner App” V2.1.2 in “TrueDepth-Mode” utilizing the device’s front-facing TrueDepth camera in a circular motion. MS PowerPoint was used to create the illustration

All scans were performed in a designated 3D scanning room designed for children with craniofacial deformities and orthognathic surgery patients. A comparative visualization of 3D imaging techniques is shown in Fig. 2.

### 3D Data processing

3D data obtained from smartphone-based methods and the Vectra M5 were exported as Wavefront OBJ files. CloudCompare's (http://cloudcompare.org/) ICP implementation was employed for rough alignment, which also included the estimation of an isotropic scaling factor. Facial areas of interest (FAOI) were extracted from both the smartphone-based SMs and Vectra-based SMs, which entailed cutting the SMs at the visible face edges. These extracted FAOIs were used for alignment, ensuring that non-facial regions and noise were excluded from the alignment process. This approach minimized the potential for errors arising from non-facial regions. Subsequently, the superimposed FAOI from the smartphone-based approaches were imported into the VAM. The VAM was utilized for precise alignment, which entailed aligning the smartphone-based FAOI with the SMs generated by the Vectra M5.

**Fig. 3:**
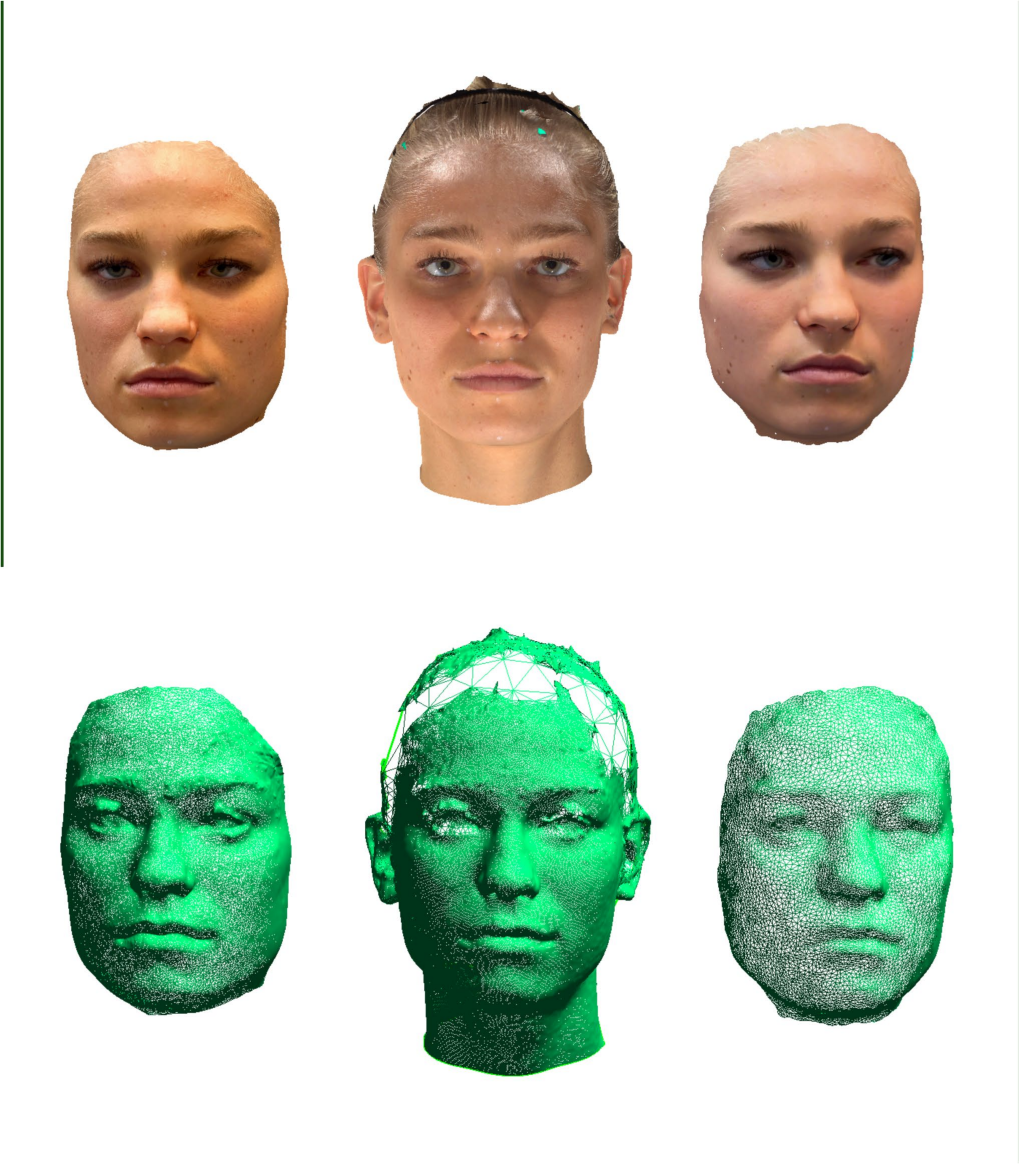
Facial areas of interest (FAOI): Appearance of a 20-year-old female participant; the iPhone 14 Pro using the application “3D Scanner App” V2.1.2 was utilized to create the SMs on the left and right side; SM generated using the “Photo-Mode” (left), Vectra M5 (middle); SM generated using the TrueDepth-Mode (right). SMs with applied texture above and without texture below

**Fig. 4:**
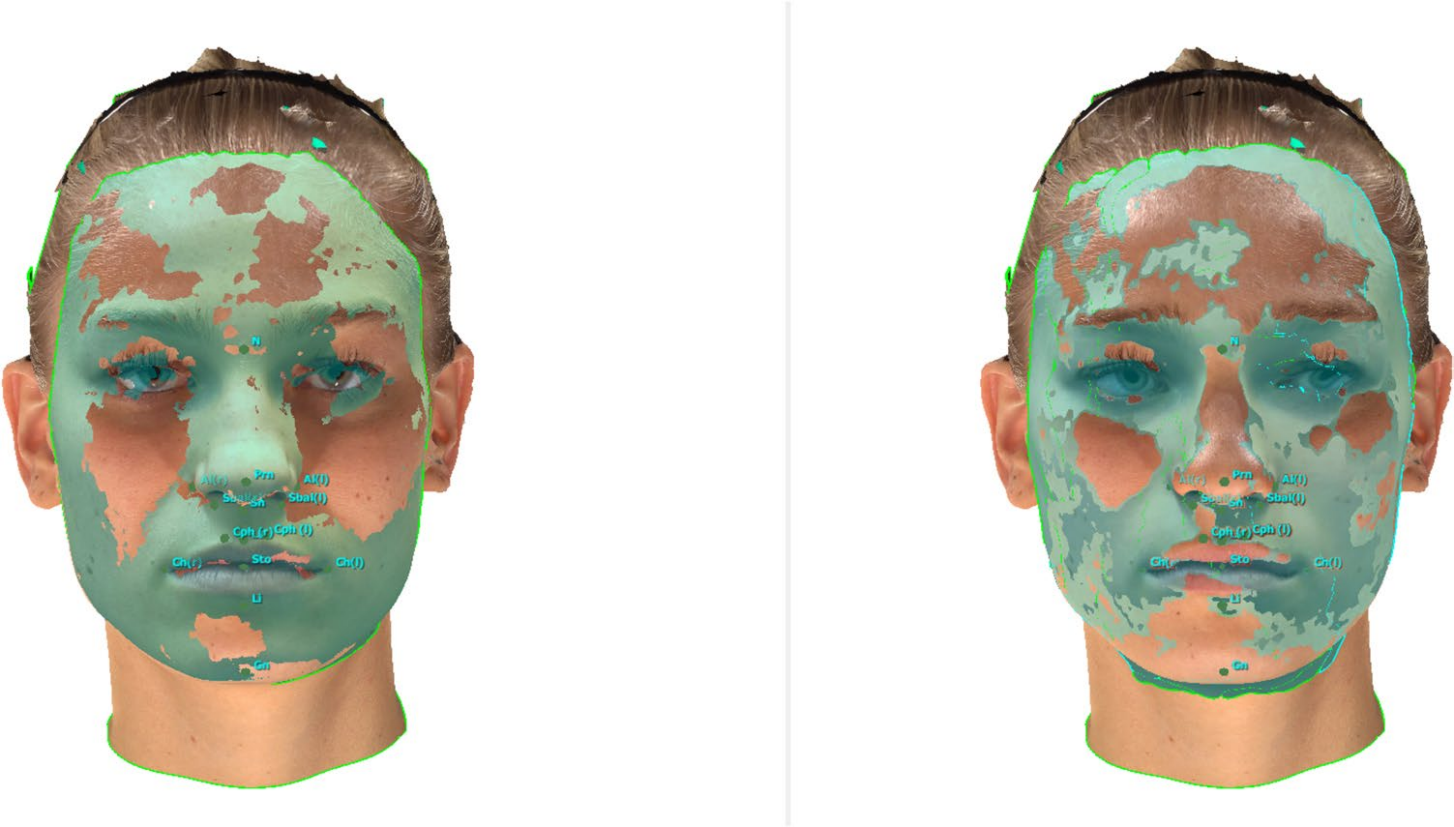
*Presentation of the superimposed SMs:* A SM generated by an iPhone 14 Pro using the “3D Scanner App’s” V2.1.2 “Photo-Mode” was superimposed onto a SM generated by the Vectra M5 (left). A SM generated by an iPhone 14 Pro using the “3D Scanner App’s” “TrueDepth-Mode” was superimposed onto a SM generated by the Vectra M5 (right)

**Fig. 5:**
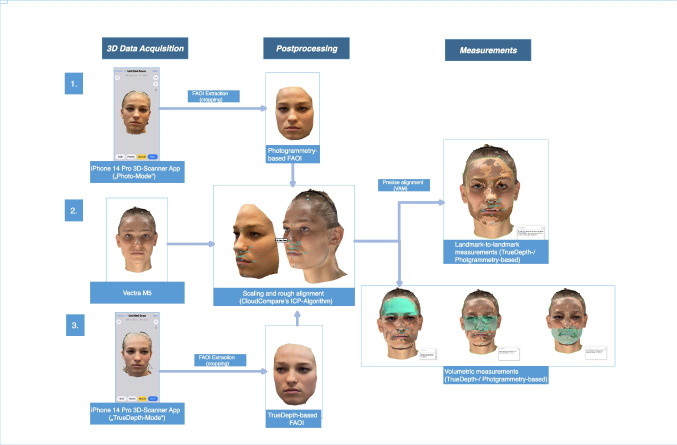
*Flowchart summarizing the study’s methodology:* 3D data acquisition, 3D data processing, and measurements. The study involved SM generation using three methods: (1) the iPhone 14 Pro’s “3D-Scanner App” V2.1.2 in “Photo-Mode” (2) stereophotogrammetry with the Vectra M5, and (3) the iPhone 14 Pro’s “3D-Scanner App” V2.1.2 in “TrueDepth-Mode”. Data processing steps included extraction of facial areas of interest (FAOIs), scaling and rough alignment via CloudCompare’s ICP-Algorithm, and superimposition and precise alignment via Vectra Analysis Module (VAM) for SM comparison. Measurements included landmark-to-landmark distances and volumetric analyses for the upper face, mid-, and lower face regions utilizing the VAM

Figure 3 juxtaposes a SM generated by the Vectra M5 to the smartphone-based SMs. Figure 4 provides an example of the superimposed SMs used for analysis. A flowchart summarizing the study’s methodology is presented in Fig. 5.

### SM-comparison

For SM comparison, the software VAM was utilized. For each participant, the study compared the TrueDepth-camera-based “3D-Scanner App” SMs with Vectra M5-based SMs and the photogrammetry-based “3D-Scanner App” SMs with Vectra M5-based SMs. FAOI derived from smartphone-based SMs were compared with Vectra-based SMs, using landmark-to-landmark distance analyses and volumetric analyses.

Landmark-to-landmark distance analyses involved assessing the surface-to-surface deviation by measuring 15 distinct landmark-to-landmark distances between the superimposed SMs. The landmarks’ midpoints were selected manually using the VAM. Subsequently, the linear distances between the superimposed models were calculated.

Volumetric analyses were conducted by generating a difference model between the superimposed SMs. This process entailed analyzing the differences in volume between the upper face, mid-face, and lower face. The upper face was defined as the area from the upper hairline to an axial plane through the nasion. The mid-face was characterized as between an axial plane through the nasion and the subnasale. The lower face was defined as between an axial plane through the subnasale and the anatomical boundaries of the lower jaw. Areas were selected manually using the VAM. Subsequently, the volumetric differences between the superimposed models were calculated for each region.

To assess inter-observer reliability, a second observer independently scaled and aligned all SMs, selected all landmarks manually, and performed all volumetric and landmark-to-landmark measurements.

**Fig. 6:**
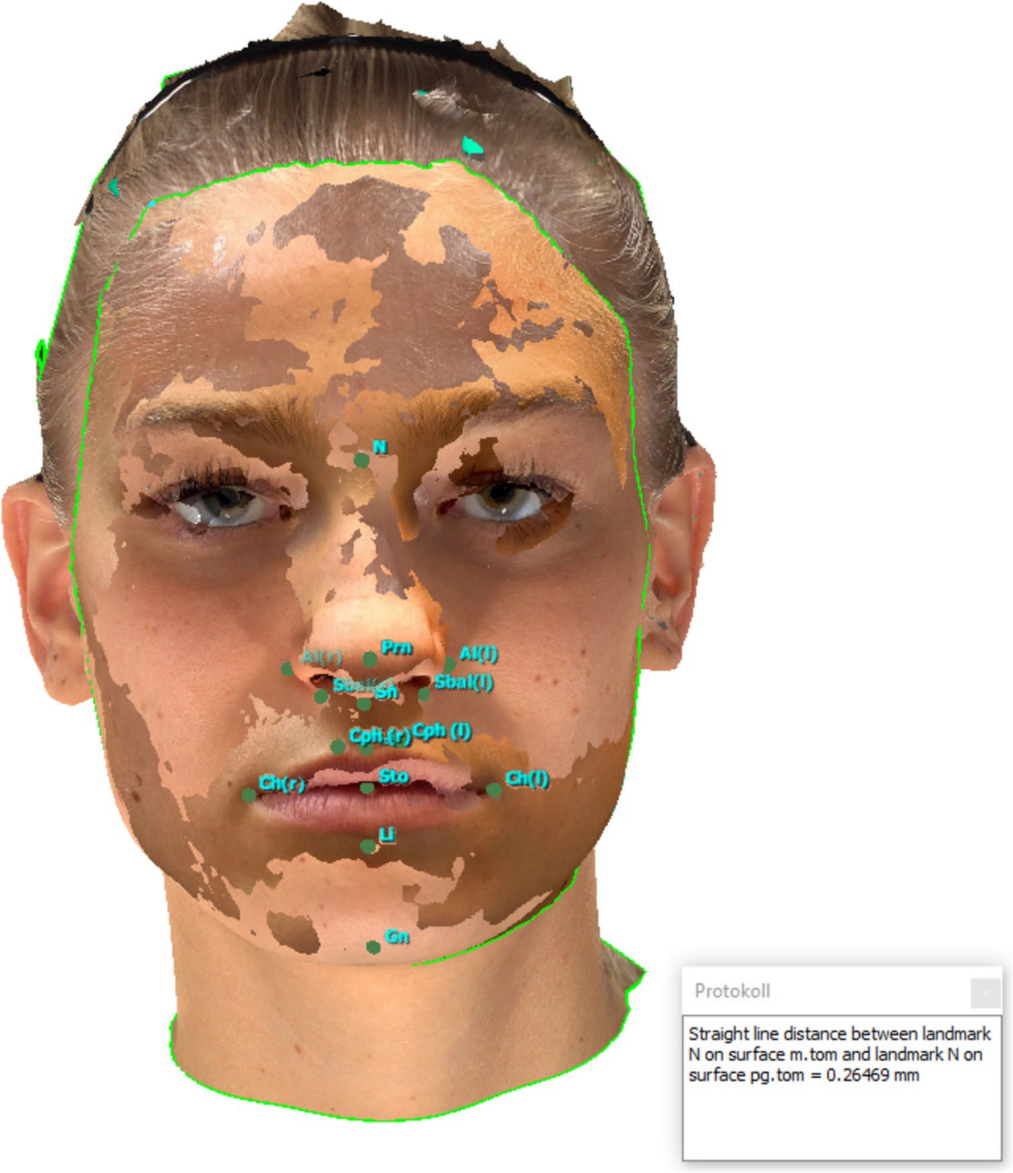
*Presentation of the landmark-to-landmark measurements:* A SM generated by an iPhone 14 Pro using the “3D Scanner App’s” V2.1.2 “Photo-Mode” was superimposed onto a SM generated by the Vectra M5; measurement (1) (Nasion – Nasion) was performed. Values in millimeters (mm)

**Fig. 7:**
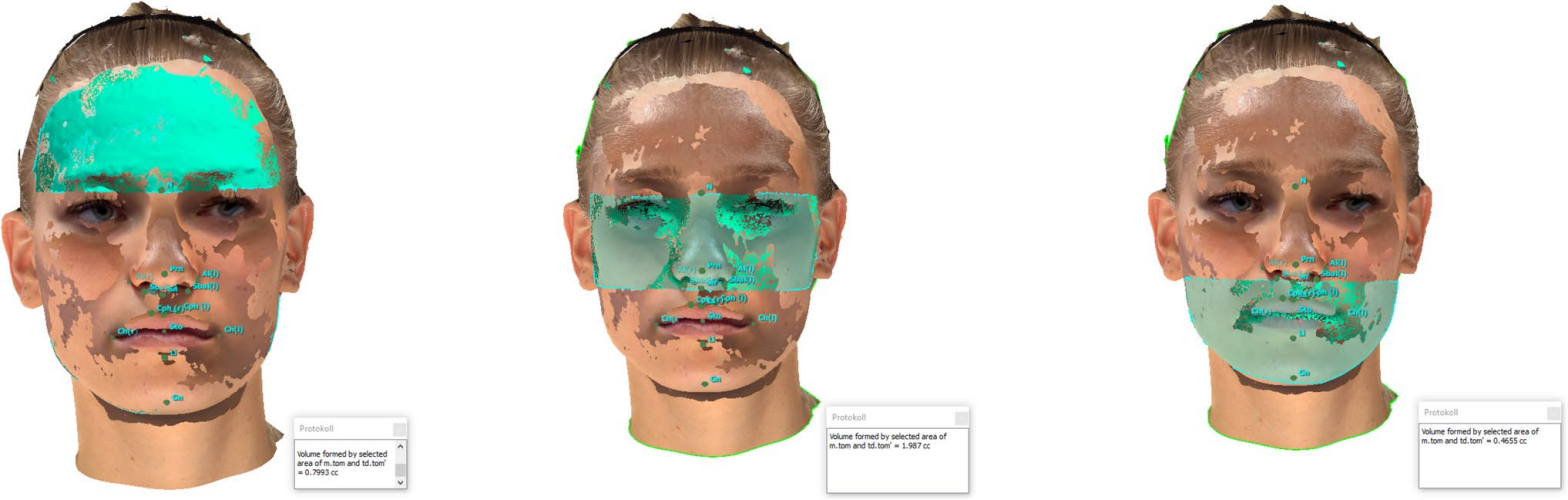
Volumetric measurements: A SM generated by an iPhone 14 Pro using the “3D Scanner App’s” V2.1.2 “TrueDepth-Mode” was superimposed onto a SM generated by the Vectra M5; Volumetric assessments of the upper face, mid-face and lower face (left to right) were performed. Values in cubic centimeters (cc)

Figure 6 provides an example of a landmark-to-landmark-distance measurement. Figure 7 shows volumetric measurements of the upper-, mid-, and lower face. Table 1 provides an overview of all measurements.

**Table 1:** Measurements performed between superimposed SMs: All measurements were performed between TrueDepth- and photogrammetry-based SMs superimposed onto Vectra-based SMs; (1) – (16) landmark-to-landmark distances; (17) – (20) volumetric distances

Measurements	
*Variables*	
*Landmark-to-landmark deviation*	*Volumetric deviation*
Distance	Distance
(1) N – N	(17) Upper face
(2) PRN – PRN	(18) Mid-face
(3) SN – SN	(19) Lower face
(4) LS – LS	(20) Overall accuracy (17) – (19)
(5) STO – STO	
(6) LI – LI	
(7) GN – GN	
(8) AL – AL (L)	
(9) AL – AL (R)	
(10) SBAL – SBAL (L)	
(11) SBAL – SBAL (R)	
(12) CPH – CPH (L)	
(13) CPH – CPH (R)	
(14) CH – CH (L)	
(15) CH – CH (R)	
(16) Overall accuracy (1) – (15)	

### Statistical analysis

IBM SPSS 29 (*SPSS Inc., USA*) was used for statistical analysis. A Shapiro–Wilk test indicated that normality could not be assumed for measurements (1) to (20). When assessing the accuracy of SMs obtained from TrueDepth- and photogrammetry-based SMs in comparison to Vectra M5-based SMs, values were considered clinically acceptable if mean values did not surpass 2 mm for landmark-to-landmark distances and 2 cc for volumetric differences. This threshold was chosen in accordance with the criteria outlined by Aung et al., who characterized measurements surpassing > 2 units from reference data as unreliable [29]. A Wilcoxon signed-rank test for paired samples was employed to compare the central tendencies between the methods. The consistency between surface-to-surface and volumetric deviation was evaluated using Bland–Altman analyses. A 95% limit of agreement (LoA) of ≤ 2 mm between TrueDepth- and photogrammetry-based SMs was defined as clinically acceptable for landmark-to-landmark distances to compare TrueDepth- and photogrammetry-based SMs based on their alignment with the Vectra M5. For volumetric deviations, a 95% Bland–Altman LoA of ≤ 2 cc was defined as clinically acceptable to compare TrueDepth- and photogrammetry-based SMs based on their alignment with the Vectra M5. Inter-observer reliability was evaluated using the Intraclass Correlation Coefficient (ICC), the Wilcoxon signed-rank test for paired samples, and Bland–Altman analyses. The ICC was evaluated according to Cicchetti et al. using the following guidelines for interpretation: less than 0.40 – poor, between 0.40 and 0.59 – fair, between 0.60 and 0.74 – good, and between 0.75 and 1.00 – excellent [30]. A 95% Bland–Altman LoA of ≤ 2 cc was considered clinically acceptable for evaluating inter-observer reliability.

## Results

### Patient demographics

The cohort included 15 men and 15 women. Their mean age was M = 24 years (SD =  ± 2.3), mean height M = 176 cm (SD =  ± 8 cm), mean weight M = 69.6 kg (SD =  ± 14.0 kg), and mean BMI M = 22.5 (SD =  ± 3.6).

### Landmark-to-Landmark Distance Analyses

#### Comparison of vectra M5- and smartphone-based SMs

Table 2 presents the outcomes of the landmark-to-landmark distance analyses.

**Table 2:** Descriptive Statistics: Landmark-to-landmark distance analyses; comparison of Vectra M5- and smartphone-based SMs; values in millimeters (mm) for TrueDepth-based and photogrammetry-based measurements (1) – (16); IBM SPSS 29 was used for data analysis

Descriptive Statistics					
Distance (mm)					
*Variables*	Vectra M5 – Photogrammetry			Vectra M5 – TrueDepth	
Distance	N	Average	SD	Average	SD
(1) N – N	30	.65	.63	.9	.75
(2) PRN – PRN	30	.55	.32	1.3	.66
(3) SN – SN	30	.60	.28	.7	.41
(4) LS – LS	30	.91	.55	.9	.68
(5) STO – STO	30	.85	.65	1.2	.65
(6) LI – LI	30	.70	.39	.8	.49
(7) GN – GN	30	.87	.49	1.2	.81
(8) AL – AL (L)	30	.64	.35	1.5	.76
(9) AL – AL (R)	30	.88	.64	1.5	.64
(10) SBAL – SBAL (L)	30	.73	.46	1.0	.57
(11) SBAL – SBAL (R)	30	.78	.49	1.2	.67
(12) CPH – CPH (L)	30	.75	.60	.9	.56
(13) CPH – CPH (R)	30	.77	.45	1.0	.64
(14) CH – CH (L)	30	1.32	1.02	1.5	.95
(15) CH – CH (R)	30	1.02	.64	1.2	.84
(16) Overall accuracy (1) – (15)	450	.8	.58	1.1	.72

The mean value for all landmark-to-landmark distances (16) of photogrammetry-based SMs to Vectra-based SMs was calculated at M = 0.8 mm (SD =  ± 0.58 mm, n = 450; Table 2). The highest deviation was found in measurement (14) (left cheilion to left cheilion) M = 1.32 mm (SD =  ± 1.02 mm, n = 30; Table 2).

The mean value for all landmark-to-landmark distances (16) between TrueDepth-based SMs and Vectra-based SMs was calculated at M = 1.1 mm (SD =  ± 0.72 mm, n = 450; Table 2). The highest deviation was found in measurement (14) (left cheilion to left cheilion) M = 1.5 mm (SD =  ± 0.95 mm, n = 30; Table 2).

All landmark-to-landmark measurements (1) – (16) remained within a clinically acceptable range, exhibiting an overall landmark-to-landmark deviation of ≤ 2 mm, when comparing both TrueDepth- and photogrammetry-based SMs with Vectra-based SMs (Table 2).

#### Comparison of truedepth- and photogrammetry-based SMs

Table 3 presents the outcomes of landmark-to-landmark distance analyses, when comparing TrueDepth- with photogrammetry-based SMs based on their alignment with Vectra-based SMs.

**Table 3:** *Landmark–to–landmark distances:* 95% Bland–Altman LoA for measurement (1) – (16); and Wilcoxon signed-rank test for paired samples for measurements (1) – (16); values in millimeters (mm); IBM SPSS 29 was used for data analysis

Bland–Altman analysis and Wilcoxon signed-rank test for paired samples							
*Variables*	Bland–Altman				Wilcoxon signed-rank test for paired samples		
	95% Confidence Interval				Median		
	N	Mean bias	Upper Bound	Lower Bound	Vectra M5 – Photogrammetry	Vectra M5 –TrueDepth	Asymp. Sig. (2-tailed)
(1) N – N	30	-.25	1.77	−2.26	.43	.64	.14
(2) PRN – PRN	30	-.8	0.51	−2.11	.45	1.27	< .001
(3) SN – SN	30	-.11	.9	−1.13	.52	.6	.35
(4) LS – LS	30	-.03	1.63	−1.7	.83	.79	.67
(5) STO – STO	30	-.35	1.30	−2.0	.68	1.16	.04
(6) LI – LI	30	-.09	1.13	−1.32	.59	.65	.40
(7) GN – GN	30	-.38	1.2	−1.95	.80	1.02	.03
(8) AL – AL (L)	30	−0.89	.84	−2.62	.61	1.47	< .001
(9) AL – AL (R)	30	-.64	1.13	−2.41	.76	1.47	.002
(10) SBAL – SBAL (L)	30	-.25	1.32	−1.82	.63	.82	.08
(11) SBAL – SBAL (R)	30	-.4	1.27	−2.06	.74	1.14	.015
(12) CPH – CPH (L)	30	-.17	1.36	−1.69	.56	.87	.09
(13) CPH – CPH (R)	30	-.23	1.12	−1.59	.62	.91	.24
(14) CH – CH (L)	30	-.16	1.89	−2.21	1.05	1.15	.28
(15) CH – CH (R)	30	-.16	1.73	−2.04	.86	1.05	.50
(16) Overall (1) – (15)	450	−0.33	1.35	−2.00	.66	.98	< .001

**Fig. 8:**
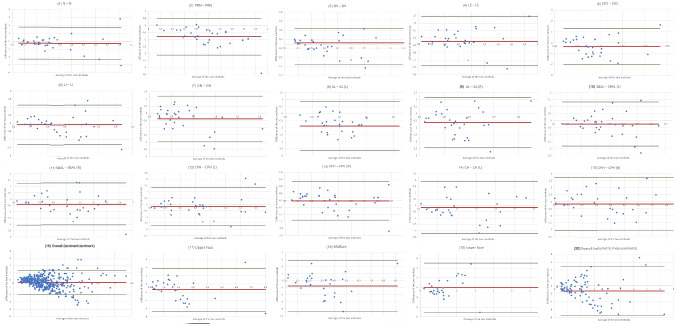
*Bland–Altman Plots:* Comparison of TrueDepth- and Photogrammetry-based SMs*.* Measurements (1) – (20); Values in millimeters (mm) for measurement (1) – (16) and cubic centimeters (cc) for measurements (17) – (20). MS Excel was used to create the illustration

Seven out of 16 measurements exceeded the clinically acceptable 95% Bland–Altman LoA of ≤ 2 mm. However, when contrasting the mean landmark-to-landmark deviation across all distances (16) of TrueDepth- and photogrammetry-based SMs, based on their alignment with Vectra-based SMs, the results indicate a clinically acceptable 95% Bland–Altman LoA of 1.35 mm to −2.0 mm (Table 3). The Wilcoxon signed-rank test for paired samples indicated that the deviation across all landmark-to-landmark distances (16) of photogrammetry-based measurements (median = 0.66 mm) was significantly lower than for TrueDepth-based measurements (median = 0.98 mm; Wilcoxon signed-rank test for paired samples; p =  < 0.001, n = 450; Table 3). Figure 8 shows the Bland–Altman plots for the landmark-to-landmark measurements (1) – (16).

### Volumetric analyses

#### Comparison of vectra M5- and smartphone-based SMs

Table 4 presents the outcomes of the volumetric deviation analyses.

**Table 4:** *Descriptive Statistics:* Volumetric difference between superimposed SMs; comparison of Vectra M5- and smartphone-based SMs; values in cubic centimeters (cc) for TrueDepth-based and photogrammetry-based measurements (17) – (20); IBM SPSS 29 was used for data analysis

Descriptive Statistics					
Volumetric difference (cc)					
*Variables*	Vectra M5 – Photogrammetry			Vectra M5 – TrueDepth	
Volumetric distance	N	Average	SD	Average	SD
(17) Upper face	30	1.60	1.91	3.0	2.47
(18) Mid-face	30	2.16	2.34	4.7	2.86
(19) Lower face	30	1.53	2.11	1.6	1.40
(20) Overall (Upper face, lower face, Midface)	90	1.8	2.12	3.1	2.64

The mean volumetric difference across all volumetric measurements (20) comparing photogrammetry-based SMs to Vectra-based SMs was calculated at M = 1.8 cc (SD =  ± 2.12 cc, n = 90). The highest deviation occurred in measurement (18) (mid-face) with M = 2.16 cc (SD =  ± 2.34 cc, n = 30; Table 4). All photogrammetry-based volumetric differences except measurement (18) (midface) remained within a clinically acceptable range, exhibiting a volumetric difference of ≤ 2 cc, when comparing photogrammetry-based SMs with Vectra-based SMs.

The mean volumetric difference across all volumetric measurements (20) for TrueDepth-based SMs compared to Vectra-based SMs was calculated at M = 3.1 cc (SD =  ± 2.64 cc, n = 90). The highest deviation was observed in measurement (18) (mid-face) with M = 4.7 cc (SD =  ± 2.86 cc, n = 30; Table 4). TrueDepth-based volumetric differences exceeded the clinically acceptable range for the overall accuracy (20), the upper- (17) and mid-face (18), exhibiting an average volumetric deviation of > 2 cc, when comparing TrueDepth-based SMs with Vectra-based SMs. However, values for the lower face (19) remained within the clinically acceptable volumetric difference of ≤ 2 cc, when comparing TrueDepth-based SMs with Vectra-based SMs (Table 4).

#### Comparison of TrueDepth- and Photogrammetry-based SMs

Table 5 presents the outcomes of volumetric deviation analyses, when comparing TrueDepth- with photogrammetry-based SMs based on their alignment with Vectra-based SMs.

**Table 5:** *Volumetric *measurements: 95% Bland–Altman LoA for measurements (17) – (20); and Wilcoxon signed-rank test for paired samples for measurements (17) – (20); values in cubic centimeters (cc); IBM SPSS 29 was used for data analysis

Bland–Altman analysis and Wilcoxon signed-rank test for paired samples							
*Variables*	Bland–Altman				Wilcoxon signed-rank test for paired samples		
			95% Confidence Interval		Median		
	N	Mean bias	Upper Bound	Lower Bound	Vectra M5 – Photogrammetry	Vectra M5 –TrueDepth	Asymp. Sig. (2-tailed)
(17) Upper face	30	-.1.4	3.85	−6.66	1.22	2.18	.01
(18) Mid-face	30	-.2.5	4.73	−9.81	1.54	4.42	< .001
(19) Lower face	30	-.03	5.05	−5.11	.74	1.23	.73
(20) Overall (Upper face, Midface, lower face)	90	−1.33	4.9	−7.6	1.14	2.12	< .001

All volumetric measurements exceeded the ≤ 2 cc 95% Bland–Altman LoA, with the highest deviation identified in the mid-face, ranging from 4.73 cc to −9.81 cc (Table 5). The Wilcoxon signed-rank test for paired samples revealed a significant difference in volumetric distances in the upper face and mid-face between the two approaches (Table 5). When contrasting the volumetric differences across all regions (20) of TrueDepth- and photogrammetry-based SMs based on their alignment with Vectra-based SMs, the results indicated a clinically unacceptable 95% Bland–Altman LoA of 4.9 cc to −7.6 cc (> 2 cc) (Table 5). The Wilcoxon signed-rank test for paired samples indicated that the deviation across all volumetric distances (20) of photogrammetry-based measurements (median = 1.14 cc) was significantly lower than for TrueDepth-based measurements (median = 2.12 cc) (Wilcoxon signed-rank test for paired samples; p =  < 0.001, n = 90; Table 5).

Figure 8 shows the Bland–Altman plots for the volumetric distances (17) – (20).

### Inter-Observer Reliability

#### Photogrammetry-based measurements

Table 6 presents the inter-observer reliability of photogrammetry-based measurements. All photogrammetry-based landmark-to-landmark measurements demonstrated good to excellent correlation, with ICC values ranging from 0.70 to 0.97. Landmark-to-landmark measurements showed a clinically acceptable 95% Bland Altman LoA of ≤ 2 mm. The Wilcoxon signed-rank test revealed no statistically significant differences between the two observers for measurements (1) – (16) (Table 6).

**Table 6:** Inter-observer reliability of photogrammetry-based measurements: Bland–Altman analysis, Wilcoxon signed-rank test and Intraclass Correlation Coefficient (ICC). Median values and mean bias in millimeters (mm) for measurements (1) – (16) and in cubic centimeters (cc) for measurements (17) – (20). OP 1 = Observer 1, OP 2 = Observer 2; IBM SPSS 29 was used for data analysis

Bland–Altman analysis, Wilcoxon signed-rank test for paired samples and ICC									ICC
*Variables*	Bland–Altman					Wilcoxon signed-rank test for paired samples			
	95% Confidence Interval				Median				
	N	Mean bias	Upper Bound	Lower Bound	OP 1		OP 2	Asymp. Sig. (2-tailed)	
(1) N – N	30	-.08	.38	-.54	.43		.55	.08	.97
(2) PRN – PRN	30	-.03	.42	-.49	.45		.49	.96	.84
(3) SN – SN	30	.04	.58	-.5	.52		.50	.54	.76
(4) LS – LS	30	-.02	.56	-.59	.83		.85	.39	.92
(5) STO – STO	30	.11	.88	-.67	.68		.60	.56	.87
(6) LI – LI	30	-.004	.47	-.48	.59		.60	.79	.9
(7) GN – GN	30	-.005	.53	-.54	.80		.65	.62	.94
(8) AL – AL (L)	30	-.07	.49	-.63	.61		.65	.14	.84
(9) AL – AL (R)	30	.06	.97	-.85	.76		.70	.94	.8
(10) SBAL – SBAL (L)	30	-.01	.52	-.55	.63		.59	.71	.91
(11) SBAL – SBAL (R)	30	-.003	.74	-.75	.74		.58	.65	.85
(12) CPH – CPH (L)	30	-.05	.97	−1.07	.56		.84	.33	.7
(13) CPH – CPH (R)	30	-.01	.62	-.64	.62		.65	.85	.89
(14) CH – CH (L)	30	.04	.94	-.86	1.05		.98	.80	.94
(15) CH – CH (R)	30	-.08	.55	-.71	.86		.90	.17	.94
(16) Overall (1) – (15)	450	-.01	.66	-.68	.66		.68	.10	.9
(17) Upper face	30	-.21	1.23	−1.66	1.22		1.36	.08	.96
(18) Mid-face	30	-.33	1.4	−2.05	1.54		1.63	.18	.96
(19) Lower face	30	-.3	1.07	−1.67	.74		.95	.17	.97
(20) Overall (Upper face, lower face, Midface)	90	-.28	1.23	−1.79	1.14		1.44	.007	.97

Volumetric assessments conducted by the two observers exhibited excellent correlation, with ICC values ranging from 0.96 to 0.97. All photogrammetry-based volumetric measurements, except for measurement (18) (midface), displayed a 95% Bland Altman LoA of ≤ 2 cc. However, the Wilcoxon signed-rank test for paired samples indicated that the deviation across all volumetric distances (20) differed significantly between the two observers (Wilcoxon signed-rank test for paired samples; p = 0.007, n = 90; Table 6).

**Fig. 9:**
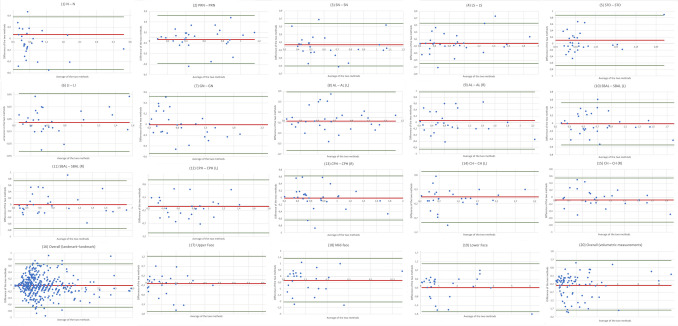
*Bland–Altman Plots:* Inter-observer reliability of photogrammetry-based measurements*.* Measurements (1) – (20); Values in millimeters (mm) for measurement (1) – (16) and cubic centimeters (cc) for measurements (17) – (20). MS Excel was used to create the illustration

Figure 9 presents the Bland–Altman plots illustrating the inter-observer reliability of photogrammetry-based measurements.

#### TrueDepth-based measurements

Table 7 presents the inter-observer reliability of TrueDepth-based measurements. The majority of landmark-to-landmark measurements ((1) – (8) and (10) – (16)) demonstrated good to excellent correlation, with ICC values ranging from 0.64 to 0.97. Measurement (9) showed fair correlation between the two observers. All landmark-to-landmark measurements displayed clinically acceptable 95% Bland–Altman LoA of ≤ 2 mm. The Wilcoxon signed-rank test revealed no statistically significant differences between the two observers for measurements (1) – (15). However, the Wilcoxon signed-rank test indicated a statistically significant difference for the deviation across all landmark-to-landmark distances (16) (Wilcoxon signed-rank test for paired samples; p < 0.001, n = 90; Table 7).

**Table 7:** Inter-observer reliability of TrueDepth-based measurements: Bland–Altman analysis, Wilcoxon signed-rank test and Intraclass Correlation Coefficient (ICC). Median values and mean bias in millimeters (mm) for measurements (1) – (16) and in cubic centimeters (cc) for measurements (17) – (20). OP 1 = Observer 1, OP 2 = Observer 2; IBM SPSS 29 was used for data analysis

Bland–Altman analysis, Wilcoxon signed-rank test for paired samples and ICC								ICC
*Variables*	Bland–Altman				Wilcoxon signed-rank test for paired samples			
	95% Confidence Interval				Median			
	N	Mean bias	Upper Bound	Lower Bound	OP 1	OP 2	Asymp. Sig. (2-tailed)	
(1) N – N	30	.07	.99	-.84	.64	.7	.71	.88
(2) PRN – PRN	30	-.06	.62	-.73	1.27	1.31	.59	.93
(3) SN – SN	30	-.16	1.07	−1.4	.6	.7	.26	.64
(4) LS – LS	30	-.05	.81	-.91	.79	.83	.25	.88
(5) STO – STO	30	-.005	.59	-.6	1.16	1.05	.59	.94
(6) LI – LI	30	-.03	.84	-.91	.65	.74	.14	.72
(7) GN – GN	30	-.001	.83	-.83	1.02	.99	.80	.92
(8) AL – AL (L)	30	-.01	.83	-.85	1.47	1.42	.58	.91
(9) AL – AL (R)	30	-.1	1.31	−1.5	1.47	1.68	.26	.57
(10) SBAL – SBAL (L)	30	-.08	.77	-.92	.82	.9	.39	.85
(11) SBAL – SBAL (R)	30	-.06	.67	-.80	1.14	1.05	.18	.92
(12) CPH – CPH (L)	30	-.11	.71	-.94	.87	.99	.08	.86
(13) CPH – CPH (R)	30	.04	1.28	−1.19	.91	.87	.48	.67
(14) CH – CH (L)	30	.01	.66	-.64	1.15	1.12	.96	.97
(15) CH – CH (R)	30	-.12	.59	-.83	1.05	1.0	.052	.95
(16) Overall (1) – (15)	450	.04	.95	-.86	.98	1.01	< .001	.97
(17) Upper face	30	-.25	3.48	−3.99	2.18	2.75	.32	.83
(18) Mid-face	30	-.01	3.05	−3.06	4.42	4.2	.35	.92
(19) Lower face	30	-.12	2.94	−3.17	1.23	1.3	.19	.51
(20) Overall (Upper face, lower face, Midface)	90	.13	3.39	−3.14	2.12	2.5	.079	.88

For volumetric assessments conducted by the two observers, excellent correlation was observed for measurements (17) (upper face), (18) (midface), and (20) (overall volume). The Wilcoxon signed-rank test revealed no statistically significant differences between the two observers for all volumetric measurements (Table 7). However, all TrueDepth-based volumetric measurements exceeded the clinically acceptable 95% Bland–Altman LoA of ≤ 2 cc between the two observers (Table 7).

**Fig. 10:**
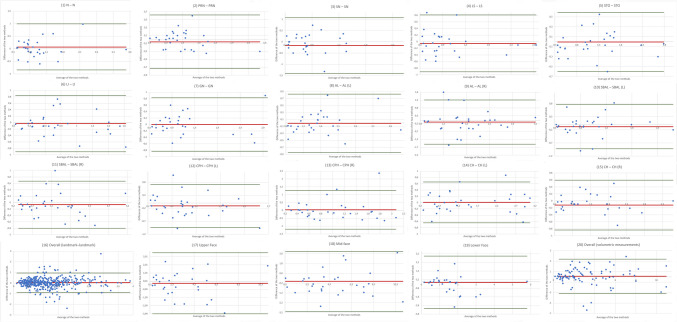
*Bland–Altman Plots:* Inter-observer reliability of TrueDepth-based measurements*.* Measurements (1) – (20); Values in millimeters (mm) for measurement (1) – (16) and cubic centimeters (cc) for measurements (17) – (20). MS Excel was used to create the illustration

Figure 10 displays the Bland–Altman plots for inter-observer reliability of TrueDepth-based measurements.

## Discussion

The present study found an overall landmark-to-landmark deviation of M = 0.8 mm (SD =  ± 0.58 mm, n = 450) for photogrammetry-based and M = 1.1 mm (SD =  ± 0.72 mm, n = 450) for TrueDepth-based SMs (Table 2). Both approaches remained within a clinically acceptable range, exhibiting an overall landmark-to-landmark deviation of ≤ 2 mm. Previous studies align with these findings, reporting a surface-to-surface deviation or landmark-to-landmark deviation of ≤ 2 mm [16, 20, 21]. The mean RMS surface-to-surface deviation for comparable systems (iPhone 11 Pro and 3dMD system) was reported at 0.86 mm ± 0.31 mm by Andrews et al. [16]. Their results indicated that midline points near the mouth and lips demonstrated less accurate results. Nightingale et al. reported an accuracy of 1.3 mm ± 0.3 mm between Artec-based and iPhone 8-based SMs [21]. Seifert et al. observed mean landmark-to-landmark deviations of 1.27 mm for the application Polycam, 1.26 mm for ScandyPro, and 1.45 mm for EM3D when comparing SMs obtained from an iPhone 14 Pro to the 3dMD. They observed that the largest deviations occurred at the stomion for all applications, with values ranging from 1.65 mm for ScandyPro to 2.02 mm for EM3D [20]. They concluded that capturing landmarks in highly flexible or variable facial regions, such as the orolabial region, poses greater challenges for smartphone-based 3D surface imaging. These findings align with the present study’s observations regarding the disparity observed in the left cheilion. Andrews et al. additionally noted that 97% of the distances between landmarks exhibited an average deviation of less than 2 mm. The current trial’s results confirm these findings, when comparing the landmark-to-landmark distances of both smartphone-based approaches to Vectra-based SMs. In contrast, Thurzo et al. observed that certain facial regions exhibited an accuracy of less than 3 mm, when assessing the accuracy of the Bellus3D Dental Pro app, utilizing the TrueDepth camera for facial 3D surface imaging [17]. In particular, the authors identified lower accuracy in deeper structures, specifically in the orbital region, consistent with the observed trend in volumetric differences in the present study. The mid-face, encompassing the orbital region, exhibited the highest volumetric deviation between Vectra M5- and smartphone-based approaches.

In this trial, the overall volumetric accuracy comparing photogrammetry-based SMs to Vectra-based SMs was calculated at M = 1.8 cc (SD =  ± 2.12 cc, n = 90; Table 4) and at M = 3.1 cc (SD =  ± 2.64 cc, n = 90; Table 4) for TrueDepth-based SMs compared to Vectra-based SMs. The overall accuracy reported in this study aligns with a study conducted by Farook et al., who found a volumetric discrepancy of 4.23 cc ± 2.28 cc comparing SMs of an ear cast obtained by the Oneplus-5t (*BBK Electronics, China*), the iPhone 6 s (*Apple Inc., USA)* and the laser scanner 3D Scanner Ultra HD (*NextEngine, USA*) [31]. However it is known, that the accuracy of anthropometric measurements may vary between smartphone applications, and the precision of SMs is influenced by the scanned object's color and shape [23, 32]. When conducting facial assessments, volumetric results may additionally be influenced by factors such as the inherent difficulty for participants to consistently maintain a neutral facial expression during 3D surface imaging [33, 34]. In addition, volumetric differences in smartphone-based 3D imaging depend on the overall measured volume, with previous investigations indicating an overall measurement error ranging from 0.67% to 3.19% [35]. Further research may contribute to a broader comprehension of smartphones' capability to anticipate volumetric alterations in the facial region.

A constraint of this study pertains to the manual extraction of FAOI, a method that may potentially limit the accuracy of the approach. However, it is essential for aligning the smartphone-based SMs with Vectra M5-based SMs. The procedure was performed consistently with previous investigations [16, 17]. Introducing automation in extracting FAOI could potentially address some of these limitations.

General limitations of 3D surface imaging must also be considered, particularly when incorporating this technology into clinical routines. A critical aspect is the standardization of lighting conditions, as they significantly impact the accuracy of smartphone-based 3D surface imaging. Light reflections can potentially affect landmark detection on the SMs [36]. To address this, the present study was conducted in a room with ambient lighting specifically designed for imaging patients with craniofacial deformities and orthognathic surgery needs. This controlled environment helped mitigate lighting-induced artifacts, a recommendation also supported by previous studies advocating the use of standardized lighting [16, 37, 38].

Another limitation is patients' inherent difficulty in maintaining a neutral facial expression during 3D surface imaging. Previous studies have shown that subtle involuntary contractions in the facial muscles can affect the accuracy of facial landmark detection and volumetric data [34, 37]. Therefore, participants were instructed to maintain a neutral facial expression throughout the imaging process. Future research could investigate stabilization methods or incorporate real-time feedback systems to assist participants in maintaining a consistent facial posture during surface imaging.

Additionally, it is noteworthy to examine the authors’ method of evaluating smartphone-based approaches in relation to their alignment with the gold standard. Aung et al. proposed that deviations exceeding 2 mm from the reference data are clinically unreliable, when comparing anthropometric measurements obtained from SMs generated by an optical surface scanner developed by the Medical Physics Department at the University College Hospital (London, UK) with direct anthropometric measurements [29]. Therefore, this study defined measurements within ≤ 2 units of the reference as clinically acceptable. When applying smartphone-based surface imaging in OMFS, it is important to consider scenarios where deviations above 2 mm or 2 cc are insufficient. These include, among others, intraoperative instrument navigation or precise preoperative planning of orthognathic surgeries, where high accuracy is required for accurate segmentation and repositioning of maxillary or mandibular segments to avoid misalignment [39]. It is important to acknowledge that smartphone-based methods can only be implemented in clinical workflows if certified as medical devices. The software used in this study was utilized in an experimental context and is not yet eligible for routine clinical application.

While smartphone-based 3D surface imaging holds significant potential, addressing variability between smartphone models and software versions is essential to ensure the reliability and generalizability of this study’s results. Several studies have reported varying levels of accuracy across different devices and software [20, 32]. Clinicians are advised to critically evaluate the accuracy of smartphone-based surface imaging software and devices before integrating them into routine clinical workflows. Future studies should focus on standardization and cross-platform validation to enhance clinical applicability.

In addition, the findings of this study regarding inter-observer reliability warrant further discussion. Photogrammetry-based measurements revealed significant differences in volumetric assessments, while TrueDepth-based measurements exceeded the 95% Bland–Altman LoA for inter-observer reliability. These discrepancies may be attributed to the study’s methodology, which required a second observer to manually relocate all landmarks, scale, and align all SMs, making it challenging to reproduce consistent volumetric measurements. This observation underscores the need for further software development to facilitate a fully automated smartphone-based approach, which could enhance reproducibility and ease of use in OMFS.

While smartphone-based 3D surface imaging may not yet fully rival the capabilities of sophisticated 3D surface imaging systems, it can function as a supplementary tool for clinicians, facilitating communication between both patients and fellow healthcare professionals. As technology advances continuously, smartphones can emerge as powerful tools for both patients and surgeons in the future.

## Conclusion

This study comprehensively examines two smartphone-based methods for facial 3D surface imaging in alignment with the current gold standard. Smartphone-based approaches using both the TrueDepth camera and photogrammetry exhibited overall landmark-to-landmark distances of ≤ 2 mm that indicated clinically acceptable results in capturing facial features compared to the Vectra M5. Photogrammetry-based SMs generated by smartphones showed higher inter-observer reliability for overall landmark-to-landmark deviation, demonstrated superior alignment, and higher volumetric accuracy with the Vectra-based SMs compared to SMs generated by the TrueDepth camera. Smartphone-based facial 3D surface imaging emerges as a potent tool for clinicians, with oral and maxillofacial surgeons leading its adoption.

## Data Availability

No datasets were generated or analysed during the current study.

## Declarations

### Clinical trial registration number (ZKS Regensburg)

Z-2024–2099-0.

## References

[1] Kau CH, Richmond S, Incrapera A, English J, Xia JJ (2007) Three-dimensional surface acquisition systems for the study of facial morphology and their application to maxillofacial surgery. Robot Comput Surg 3:97–110. 10.1002/rcs.141 10.1002/rcs.141 17619242

[2] Tzou C-HJ, Frey M (2011) Evolution of 3D Surface Imaging Systems in Facial Plastic Surgery. Facial Plastic Surgery Clinics of North America 19:591–602. 10.1016/j.fsc.2011.07.003 22004854 10.1016/j.fsc.2011.07.003

[3] Nguyen C, Nicolai ESJ, He JJ, Roshchupkin GV, Corten EML (2022) 3D surface imaging technology for objective automated assessment of facial interventions: A systematic review. J Plast Reconstr Aesthet Surg 75:4264–4272. 10.1016/j.bjps.2022.06.086 36127225 10.1016/j.bjps.2022.06.086

[4] Lekakis G, Claes P, Hamilton G, Hellings P (2016) Three-Dimensional Surface Imaging and the Continuous Evolution of Preoperative and Postoperative Assessment in Rhinoplasty. Facial Plast Surg 32:088–094. 10.1055/s-0035-1570122 10.1055/s-0035-1570122 26862969

[5] Alfertshofer M, Frank K, Melnikov DV, Möllhoff N, Gotkin RH, Freytag DL et al (2021) Performing Distance Measurements in Curved Facial Regions: A Comparison between Three-Dimensional Surface Scanning and Ultrasound Imaging. Facial Plast Surg 37:395–399. 10.1055/s-0041-1725166 33706385 10.1055/s-0041-1725166

[6] Jayaratne YSN, Zwahlen RA (2014) Application of Digital Anthropometry for Craniofacial Assessment. Craniomaxillofac Trauma Reconstr 7:101–107. 10.1055/s-0034-1371540 25050146 10.1055/s-0034-1371540 PMC4078131

[7] Sforza C, Ulaj E, Gibelli DM, Allevi F, Pucciarelli V, Tarabbia F et al (2018) Three-dimensional superimposition for patients with facial palsy: an innovative method for assessing the success of facial reanimation procedures. Br J Oral Maxillofac Surg 56:3–7. 10.1016/j.bjoms.2017.11.015 29223635 10.1016/j.bjoms.2017.11.015

[8] Yamada T, Mishima K, Moritani N, Janune D, Matsumura T, Ikeya Y et al (2010) Nasolabial Morphologic Changes After a Le Fort I Osteotomy: A Three-Dimensional Anthropometric Study. J Craniofac Surg 21:1089–1095. 10.1097/SCS.0b013e3181e1e6ff 20613575 10.1097/SCS.0b013e3181e1e6ff

[9] Tzou C-HJ, Artner NM, Pona I, Hold A, Placheta E, Kropatsch WG et al (2014) Comparison of three-dimensional surface-imaging systems. J Plast Reconstr Aesthet Surg 67:489–97. 10.1016/j.bjps.2014.01.003 24529695 10.1016/j.bjps.2014.01.003

[10] Van Der Vlis M, Dentino KM, Vervloet B, Padwa BL (2014) Postoperative Swelling After Orthognathic Surgery: A Prospective Volumetric Analysis. J Oral Maxillofac Surg 72:2241–2247. 10.1016/j.joms.2014.04.026 25236819 10.1016/j.joms.2014.04.026

[11] Alhazmi B, Alshomer F, Alazzam A, Shehabeldin A, Almeshal O, Kalaskar DM (2022) Digital workflow for fabrication of bespoke facemask in burn rehabilitation with smartphone 3D scanner and desktop 3D printing: clinical case study. 3D Print Med 8:12. 10.1186/s41205-022-00140-0 35507199 10.1186/s41205-022-00140-0 PMC9069819

[12] Weissler JM, Stern CS, Schreiber JE, Amirlak B, Tepper OM (2017) The Evolution of Photography and Three-Dimensional Imaging in Plastic Surgery. Plast Reconstr Surg 139:761–769. 10.1097/PRS.0000000000003146 28234862 10.1097/PRS.0000000000003146

[13] D ‘Ettorre G, Farronato M, Candida E, Quinzi V, Grippaudo C (2022) A comparison between stereophotogrammetry and smartphone structured light technology for three-dimensional face scanning. Angle Orthod 92:358–63. 10.2319/040921-290.1 10.2319/040921-290.1 PMC9020391 35015071

[14] Akan B, Akan E, Şahan AO, Kalak M (2021) Evaluation of 3D Face-Scan images obtained by stereophotogrammetry and smartphone camera. Int Orthod 19:669–678. 10.1016/j.ortho.2021.08.007 34544662 10.1016/j.ortho.2021.08.007

[15] Rudy HL, Wake N, Yee J, Garfein ES, Tepper OM (2020) Three-Dimensional Facial Scanning at the Fingertips of Patients and Surgeons: Accuracy and Precision Testing of iPhone X Three-Dimensional Scanner. Plast Reconstr Surg 146:1407–1417. 10.1097/PRS.0000000000007387 33234980 10.1097/PRS.0000000000007387

[16] Andrews J, Alwafi A, Bichu YM, Pliska BT, Mostafa N, Zou B (2023) Validation of three-dimensional facial imaging captured with smartphone-based photogrammetry application in comparison to stereophotogrammetry system. Heliyon 9:e15834. 10.1016/j.heliyon.2023.e15834 37180897 10.1016/j.heliyon.2023.e15834 PMC10172784

[17] Thurzo A, Strunga M, Havlínová R, Reháková K, Urban R, Surovková J et al (2022) Smartphone-Based Facial Scanning as a Viable Tool for Facially Driven Orthodontics? Sensors 22:7752. 10.3390/s22207752 36298103 10.3390/s22207752 PMC9607180

[18] Chong Y, Liu X, Shi M, Huang J, Yu N, Long X (2021) Three-dimensional facial scanner in the hands of patients: validation of a novel application on iPad/iPhone for three-dimensional imaging. Ann Transl Med 9:1115–1115. 10.21037/atm-21-1620 34430556 10.21037/atm-21-1620 PMC8350646

[19] Quinzi V, Polizzi A, Ronsivalle V, Santonocito S, Conforte C, Manenti RJ et al (2022) Facial Scanning Accuracy with Stereophotogrammetry and Smartphone Technology in Children: A Systematic Review. Children 9:1390. 10.3390/children9091390 36138698 10.3390/children9091390 PMC9498045

[20] Seifert LB, Beyer M, Czok V, Aigner A, Abazi S, Thieringer FM et al (2024) Comparative Accuracy of Stationary and Smartphone-Based Photogrammetry in Oral and Maxillofacial Surgery: A Clinical Study. JCM 13:6678. 10.3390/jcm13226678 39597822 10.3390/jcm13226678 PMC11594577

[21] Nightingale RC, Ross MT, Allenby MC, Woodruff MA, Powell SK (2020) A Method for Economical Smartphone-Based Clinical 3D Facial Scanning. J Prosthodont 29:818–825. 10.1111/jopr.13274 33089546 10.1111/jopr.13274

[22] Othman SA, Saffai L, Wan Hassan WN (2020) Validity and reproducibility of the 3D VECTRA photogrammetric surface imaging system for the maxillofacial anthropometric measurement on cleft patients. Clin Oral Invest 24:2853–2866. 10.1007/s00784-019-03150-1 10.1007/s00784-019-03150-1 31754872

[23] Vogt M, Rips A, Emmelmann C (2021) Comparison of iPad Pro®’s LiDAR and TrueDepth Capabilities with an Industrial 3D Scanning Solution. Technologies 9:25. 10.3390/technologies9020025

[24] De Stefani A, Barone M, Hatami Alamdari S, Barjami A, Baciliero U, Apolloni F et al (2022) Validation of Vectra 3D Imaging Systems: A Review. IJERPH 19:8820. 10.3390/ijerph19148820 35886670 10.3390/ijerph19148820 PMC9318949

[25] Nieberle F, Spoerl S, Lottner L-M, Spanier G, Schuderer JG, Fiedler M et al (2023) Direct Anthropometry Overestimates Cranial Asymmetry—3D Digital Photography Proves to Be a Reliable Alternative. Diagnostics 13:1707. 10.3390/diagnostics13101707 37238192 10.3390/diagnostics13101707 PMC10216941

[26] Othman SA, Aidil Koay NA (2016) Three-dimensional facial analysis of Chinese children with repaired unilateral cleft lip and palate. Sci Rep 6:31335. 10.1038/srep31335 27507713 10.1038/srep31335 PMC4979089

[27] Douglas TS (2004) Image processing for craniofacial landmark identification and measurement: a review of photogrammetry and cephalometry. Comput Med Imaging Graph 28:401–409. 10.1016/j.compmedimag.2004.06.002 15464879 10.1016/j.compmedimag.2004.06.002

[28] Santander P, Quast A, Hubbert J, Horn S, Meyer-Marcotty P, Küster H et al (2020) Three-dimensional head shape acquisition in preterm infants - Translating an orthodontic imaging procedure into neonatal care. Early Human Dev 140:104908. 10.1016/j.earlhumdev.2019.104908 10.1016/j.earlhumdev.2019.104908 31670175

[29] Aung SC, Ngim RCK, Lee ST (1995) Evaluation of the laser scanner as a surface measuring tool and its accuracy compared with direct facial anthropometric measurements. Br J Plast Surg 48:551–558. 10.1016/0007-1226(95)90043-8 8548155 10.1016/0007-1226(95)90043-8

[30] Cicchetti DV (1994) Guidelines, Criteria, and Rules of Thumb for Evaluating Normed and Standardized Assessment Instruments in Psychology. Psychol Assess 6(4):284

[31] Farook TH, Rashid F, Jamayet NB, Abdullah JY, Dudley J, Khursheed AM (2022) A virtual analysis of the precision and accuracy of 3-dimensional ear casts generated from smartphone camera images. J Prosthet Dent 128:830–836. 10.1016/j.prosdent.2020.12.041 33642077 10.1016/j.prosdent.2020.12.041

[32] Van Lint L, Christiaens L, Stroo V, Bila M, Willaert R, Sun Y et al (2023) Accuracy Comparison of 3D Face Scans Obtained by Portable Stereophotogrammetry and Smartphone Applications. J Med Biol Eng 43:550–560. 10.1007/s40846-023-00817-9

[33] Aynechi N, Larson BE, Leon-Salazar V, Beiraghi S (2011) Accuracy and precision of a 3D anthropometric facial analysis with and without landmark labeling before image acquisition. Angle Orthod 81:245–252. 10.2319/041810-210.1 21208076 10.2319/041810-210.1 PMC8925260

[34] Rawlani R, Qureshi H, Rawlani V, Turin SY, Mustoe TA (2019) Volumetric Changes of the Mid and Lower Face with Animation and the Standardization of Three-Dimensional Facial Imaging. Plast Reconstr Surg 143:76–85. 10.1097/PRS.0000000000005082 30589778 10.1097/PRS.0000000000005082

[35] Saif W, Alshibani A. Smartphone-Based Photogrammetry Assessment in Comparison with a Compact Camera for Construction Management Applications 2022.

[36] Ghoddousi H, Edler R, Haers P, Wertheim D, Greenhill D (2007) Comparison of three methods of facial measurement. Int J Oral Maxillofac Surg 36:250–258. 10.1016/j.ijom.2006.10.001 17113754 10.1016/j.ijom.2006.10.001

[37] Heike CL, Upson K, Stuhaug E, Weinberg SM (2010) 3D digital stereophotogrammetry: a practical guide to facial image acquisition. Head Face Med 6:18. 10.1186/1746-160X-6-18 20667081 10.1186/1746-160X-6-18 PMC2920242

[38] Lane C, Harrell W (2008) Completing the 3-dimensional picture. Am J Orthod Dentofac Orthop 133:612–620. 10.1016/j.ajodo.2007.03.023 10.1016/j.ajodo.2007.03.023 18405826

[39] Wilde F, Schramm A (2013) Rekonstruktion nach Trauma: Bedeutung moderner Bildgebung und Assistenzverfahren. MKG-Chirurg 6:154–164. 10.1007/s12285-012-0336-5

